# Long-term Impact of Temporal Sequence from Childhood Obesity to Hyperinsulinemia on Adult Metabolic Syndrome and Diabetes: The Bogalusa Heart Study

**DOI:** 10.1038/srep43422

**Published:** 2017-02-23

**Authors:** Tao Zhang, Huijie Zhang, Ying Li, Shengxu Li, Camilo Fernandez, Lydia Bazzano, Jiang He, Fuzhong Xue, Wei Chen

**Affiliations:** 1 Department of Biostatistics, School of Public Health, Shandong University, Jinan, China; 2 Department of Epidemiology, School of Public Health and Tropical Medicine, Tulane University, New Orleans, LA, USA; 3 Department of Endocrinology and Metabolism, the First Hospital of Xiamen, Xiamen University, Xiamen, China; 4 Department of Nutrition and Food Hygiene, School of Public Health, Harbin Medical University, Harbin, China

## Abstract

This study aims to delineate the temporal relations between body mass index (BMI) and insulin in childhood and their impact on adult metabolic syndrome (MetS) and type 2 diabetes mellitus (T2DM).The longitudinal cohort consisted of 609 whites and 339 blacks who had BMI and fasting insulin measured twice in childhood (mean age = 10.5 years at baseline and 15.9 years at follow-up). Incident MetS and T2DM were identified in adulthood (mean age = 30.5 years). Cross-lagged panel and mediation analysis models were used. After adjusting for age, race, gender, and follow-up years, the cross-lagged path coefficient of BMI → insulin (β = 0.326, p < 0.001) was significantly greater than that of insulin → BMI (β = −0.023, p = 0.207) in childhood, with p < 0.001 for the difference in βs. The path coefficient for BMI → insulin was significantly greater in MetS than in non-MetS groups (0.510 vs 0.190, p < 0.001), and greater in hyperglycemia than in normoglycemia groups (0.503 vs 0.285, p = 0.026). The mediation effect of childhood insulin on the BMI-MetS and BMI-hyperglycemia associations was estimated at 19.2% (p < 0.001) and 18.3% (p < 0.001), respectively. These findings provide evidence that higher BMI levels precede hyperinsulinemia during childhood, and this one-directional relation plays a significant role in the development of MetS and T2DM in adult life.

Despite enormous efforts of research and prevention over the past few decades, there is still an upward trend worldwide in the prevalence of obesity, metabolic syndrome (MetS) and type 2 diabetes mellitus (T2DM)^1^,^2^. These metabolic disorders are well known risk factors of cardiovascular disease^3^,^4^,^5^. Obesity and insulin resistance are thought to be primary antecedent abnormalities in the development of MetS and T2DM^4^,^5^,^6^,^7^,^8^,^9^,^10^. With the soaring trajectory of childhood obesity, MetS and T2DM are now being diagnosed in an ever-increasing number of youth^11^. To “halt the rise in diabetes and obesity” in adults and children was one of the global health targets set by the World Health Assembly in 2013^12^.

The notion of “childhood origins” of MetS and T2DM is supported by numerous publications from population-based cohorts followed since childhood, including the Bogalusa Heart Study^6^,^9^,^13^,^14^,^15^. There is a huge body of evidence showing that the strong inter-correlation between obesity and insulin resistance plays a crucial role in the development of MetS and T2DM. Our previous studies have demonstrated that long-term impact of obesity on MetS and T2DM is modified by insulin resistance in the longitudinal cohort of children and young adults^15^,^16^,^17^. We also delineated the temporal sequence from obesity to insulin resistance by providing evidence that higher body mass index (BMI) levels precede hyperinsulinemia during childhood^18^. Although childhood BMI and insulin levels are extensively reported to be associated with MetS and T2DM in later life, how their causal relation patterns in childhood influence adult MetS and T2DM, and to what extent obesity is associated with MetS and T2DM through insulin resistance are largely unknown.

The cross-lagged analysis model is typically to dissect the temporal sequences of inter-correlated variables measured at two time points in a longitudinal study and help construct the mediation analysis model. Utilizing a longitudinal cohort from the Bogalusa Heart Study, the present study aims to examine the temporal sequence between childhood BMI and insulin using cross-lagged panel analysis and explore the impact of their temporal relationship patterns on adult MetS and T2DM using mediation analysis models.

## Results

Table 1 summarizes mean levels (standard deviation) of study variables in childhood at baseline and follow-up, and adulthood by race and gender. The mean levels of continuous variables were compared between race and gender groups, adjusting for age (except age itself). In general, BMI and insulin showed significant race difference (blacks > whites) in childhood follow-up survey and adulthood, especially in females. Adulthood systolic blood pressure (SBP, blacks > whites, males > females), high-density lipoprotein cholesterol (HDL-C, blacks > whites) and triglycerides (whites > blacks, males > females) had significant race and gender differences. The prevalence of MetS, impaired fasting glucose (IFG) and T2DM did not show significant race and gender differences.

Supplement Table S1 presents pair-wise Pearson correlations between childhood baseline and follow-up values of BMI and insulin in the total sample and by race, MetS and hyperglycemia groups, adjusted for covariates where appropriate. The correlation coefficients between baseline BMI and follow-up insulin differed significantly between race, MetS, T2DM and hyperglycemia groups.

**Figure 1:**
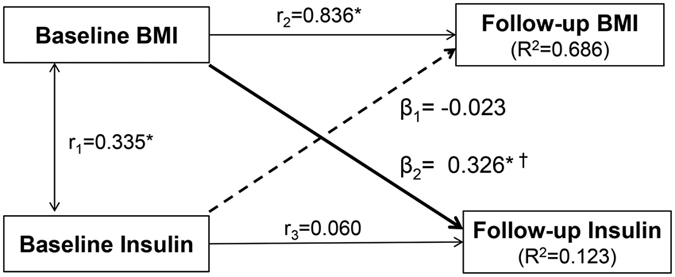
Cross-lagged path analysis of childhood BMI and insulin, adjusted for age, race, gender, and follow-up years. β_1_, β_2_ = cross-lagged path coefficients; r_1_ = synchronous correlations; r_2_, r_3_ = tracking correlations; R^2^ = variance explained. Goodness-of-fit: RMR = 0.062 and CFI = 0.904. Coefficients different from 0: *p < 0.01, ^†^p < 0.001 for difference between β_1_ and β_2_.

Figure 1 presents the cross-lagged path analysis of childhood BMI and insulin. After adjusting for age, race, gender and follow-up years, the path coefficient from baseline BMI to follow-up insulin (β_2_ = 0.326, *p* < 0.001) was significantly greater than the path coefficient from baseline insulin to follow-up BMI (β_1_ = −0.023, *p* = 0.207), with *p* < 0.001 for difference between β_1_ and β_2_. Autocorrelation (r_2_) also known as tracking correlation of BMI was significantly greater than that of insulin (r_3_). The variance (R^2^) of follow-up BMI explained by baseline predictors was greater than that of follow-up insulin. Root mean square residual (RMR) and comparative fit index (CFI) were 0.062 and 0.904, respectively, indicating a relatively good fit to the observed data according to the criteria of RMR < 0.05 and CFI > 0.90.

Table 2 presents cross-lagged path coefficients by race, MetS and hyperglycemia groups, adjusted for age, race, gender, and follow-up years. Although the difference between β_1_ and β_2_ was significant (p < 0.001) within each race group, the path coefficients (β_1_ and β_2_) did not show significant difference between blacks and whites (p = 0.735 for β_1_ and p = 0.207 for β_2_). Similar patterns of the difference between β_1_ and β_2_ were noted in MetS and hyperglycemia groups. The path coefficient (β_1_) from baseline insulin to follow-up BMI did not differ significantly between MetS and non-MetS groups (p = 0.212) as well as between normoglycemic and hyperglycemic groups (p = 0.966). The path coefficient (β_2_) from baseline BMI to follow-up insulin was significantly greater in the MetS group than in non-MetS group (0.510 vs 0.190, p < 0.001) and in the hyperglycemic group than normoglycemic group (0.503 vs 0.285, p = 0.026). Model fitting parameters ranged from 0.018 to 0.065 for RMR and from 0.895 to 0.998 for CFI. Detailed parameter information on cross-lagged path analysis models of childhood BMI and insulin in MetS and hyperglycemia groups were provided in Supplement Figures S1 and S2.

Since there were wide ranges of the number of follow-up years during childhood and from childhood to adulthood, the path coefficients (β_1_ and β_2_) were estimated in tertile groups of number of follow-up years. β_1_ and β_2_ did not differ significantly between tertile subgroups of follow-up years during childhood (Supplement Table S2) and from the last childhood survey to adulthood (Supplement Table S3).

**Figure 2:**
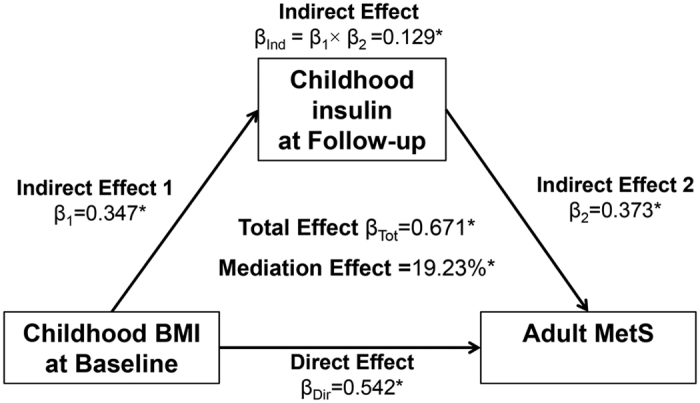
Mediation effect of childhood insulin on the childhood BMI-adult MetS association.

**Figure 3:**
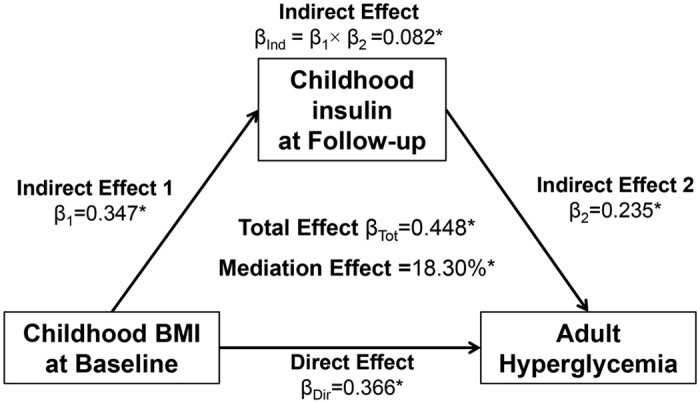
Mediation effect of childhood insulin on the childhood BMI-adult hyperglycemia association.

Figures 2 and 3 present the mediation effect of childhood follow-up insulin on the associations of childhood BMI with adult MetS and hyperglycemia, adjusted for age, race, gender and follow-up years. The mediation effect of childhood follow-up insulin was 19.2% (p < 0.001) on the childhood BMI-adult MetS association and 18.3% (p < 0.001) on the childhood BMI-adult hyperglycemia association.

## Discussion

The present study examined the temporal relationship between BMI and insulin levels in a longitudinal cohort of black and white children using a cross-lagged path analysis model, a statistical approach to dissecting a causal relationship between inter-correlated variables^19^,^20^. The results indicated that higher BMI at baseline preceded higher insulin levels at follow-up during childhood, with blacks and whites showing similar patterns of this one-directional relationship. The findings from this and our previous studies in children^18^,^21^ support the notion that the temporal nature of the dynamics of the relation between obesity and hyperinsulinemia begins in early life. Obesity and insulin resistance are well-established risk factors for MetS and T2DM in children and adults^3^,^4^,^10^,^22^. Despite the general concept that insulin resistance is a key link of obesity with MetS and T2DM, convincing evidence is still lacking from population studies regarding the causal sequence (temporal relationship) between obesity and insulin resistance with compensatory hyperinsulinemia. The Bogalusa Heart Study has attempted to demonstrate the temporal relation between BMI and insulin over a 3-year follow-up during childhood, adolescents and young adults; but the results were not conclusive, especially in adolescents^21^. The Normative Aging Study reported that the “chicken-egg” question of the dynamics of the relation between insulin levels and obesity is far from straightforward because changes in either one may precede changes in the other using the four successive examinations with mean intervals of 3.7, 3.3, and 3.0 years in adults^23^. Previous studies have used traditional longitudinal analysis models which cannot specifically address the causal relation.

It is generally considered that obesity measures and insulin levels can influence each other based on pathophysiological and metabolic mechanisms^24^. The adipose tissue is crucial in regulating insulin sensitivity through increased adipocyte size, decreased numbers of insulin receptors, increased levels of circulating free fatty acids, abnormal adipokines like decreased adiponectin and the accumulation of lipids in target tissues (lipotoxicity)^24^,^25^,^26^. On the other hand, there are mechanisms by which hyperinsulinemia could lead to obesity through affecting dietary intake (carbohydrate craving) or other mechanisms^26^,^27^,^28^. Furthermore, there is overwhelming evidence for the effect of insulin therapy on body weight increase in diabetic patients. Several explanations for the mechanisms by which intense insulin treatment causes weight gain have been suggested, including hyperphagia following hypoglycemic stimuli, alteration of physical activity level, the anabolic and/or lipogenic actions of insulin, and/or decreased glycosuria^29^,^30^,^31^,^32^,^33^,^34^,^35^. The current study found that baseline hyperinsulinemia did not lead to follow-up obesity in childhood. These observations suggest that the mechanisms for the insulin therapy-induced obesity in diabetic patients might be different from those for the association between obesity and hyperinsulinemia as a compensatory indictor of insulin resistance in the general population.

The current study provided evidence for the early origin hypothesis of MetS and T2DM by demonstrating that the one-directional relationship from obesity to hyperinsulinemia in childhood was significantly stronger in MetS and hyperglycemia groups than in non-MetS and normoglycemia groups in adulthood. Insulin resistance is considered a key link of obesity with MetS and T2DM based on pathophysiological and metabolic mechanisms^3^,^7^,^36^. The joint or synergistic effect of obesity and insulin resistance on the development of MetS and T2DM has been documented in previous studies^15^,^17^; however, data are lacking regarding the mediation effect of insulin resistance on the association of obesity with MetS and T2DM. In the present study, the BMI-to-insulin directionality in childhood was established in the cross-lagged analysis models, and then the temporal patterns were linked to adult MetS and T2DM developed 14.6 years later since the last childhood survey.

The results of causal mediation analysis of the present study showed that although the childhood BMI-adult MetS and childhood BMI-adult hyperglycemia associations were partially mediated by childhood hyperinsulinemia, the mediation effect was much smaller than the direct effect of childhood BMI on adult MetS and hyperglycemia. The findings of this study suggest that hyperinsulinemia is only one of the mediators in the pathway from obesity to T2DM; there exist other important underlying pathophysiological and metabolic mechanisms involved in the direct effect of childhood obesity on MetS and T2DM. No data in this regard are available for comparison; further studies are needed to validate the findings from the current study.

This community-based longitudinal cohort study provides a unique opportunity to examine temporal relationships between childhood BMI and insulin and their impact on the incident adult MetS and T2DM; however, it has certain limitations. First, the causal inference and causal mediation analyses were linked in the current study, with childhood follow-up insulin as a single mediator. As discussed above, there are multiple mechanisms underlying the obesity-T2DM association in addition to insulin resistance. More sophisticated mediation analysis models with multiple mediators need to be attempted in this regard. Second, the number of follow-up years of this longitudinal cohort ranged 2.0~14.7 years during childhood and 3.0~30.2 years from the last childhood to adult survey. The wide range of follow-up time might have an influence on the cross-lagged analysis parameters and their association with adult outcomes. For this reason, we conducted additional association analyses by tertile groups of the follow-up years and did not find a significant difference in parameters of the cross-lagged and association analyses among three subgroups as shown in Supplement Tables S2 and S3. Third, the small sample size of blacks had a relatively limited power to detect the weak-to-moderate associations.

In summary, the current study demonstrated that increased BMI levels preceded hyperinsulinemia in childhood in longitudinal assessment of their directionality using cross-lagged path analysis model. Importantly, this one-directional relation was much stronger in MetS and hyperglycemia groups than in non-MetS and normoglycemia groups diagnosed in adulthood. Significant mediation effects of childhood insulin on the association of childhood BMI with adult MetS and T2DM were demonstrated. The findings of the causal inference analysis of childhood BMI and insulin in relation to adult MetS and T2DM would improve our understanding of the pathobiology, mechanisms and natural history of human metabolic risk profiles, and facilitate selection of novel therapeutic and intervention strategies by targeting at the causal factors, especially early in life, to prevent future metabolic disorders and cardiovascular diseases.

## Methods

### Study Cohort

The Bogalusa Heart Study is a series of long-term studies in a semi-rural biracial (65% white and 35% black) community in Bogalusa, Louisiana since in 1973 founded by Dr. Gerald Berenson. This study focuses on the early natural history of cardiovascular disease from childhood^14^. Between 1973 and 2010, nine cross-sectional surveys of children ages 4 to 18 years and ten cross-sectional surveys of adults, aged 19 to 52 years, who had been previously examined as children were conducted in Bogalusa. This panel design of repeated cross-sectional examinations has resulted in serial observations every 2 to 3 years from childhood to adulthood. The longitudinal cohort of this study consisted of 948 adult subjects (609 whites and 339 blacks; 39.1% males; mean age = 30.5 years with a range of 20.1~49.2 years in adulthood). These adults were examined twice for BMI and insulin levels 5.4 years apart in childhood (mean age = 10.5 years at baseline and 15.9 years at follow-up). The follow-up period was 14.6 years on average (range = 3.0~30.2 years) from the last childhood to adult survey. There were no diabetes patients in this study cohort during childhood. Incident T2DM was identified in adulthood as the outcome.

All subjects in this study gave informed consent for each survey, and for those under 18 years of age, consent of a parent/guardian was obtained. Study protocols were approved by the Institutional Review Board of the Tulane University Health Sciences Center. The methods were carried out in accordance with the approved guidelines.

### Measurements

Standardized protocols were used by trained examiners across all surveys since 1973^14^. Subjects were instructed to fast for 12 hours before screening. Replicate measurements of height and weight were made, and the mean values were used for analysis. BMI (weight in kilograms divided by the square of the height in meters) was used as a measure of overall adiposity. The waist circumference (WC) was measured midway between the rib cage and the superior border of the iliac crest while subjects were standing. Two measurements were obtained with a non-stretchable tape, and the mean value was used for analysis. Blood pressure levels were measured between 8:00 AM and 10:00 AM on the right arm of subjects in a relaxed, sitting position by 2 trained observers (3 replicates each). SBP and DBP were recorded using a mercury sphygmomanometer. The mean values of the 6 readings were used for analysis.

Serum triglycerides levels were assayed using enzymatic procedures on a Hitachi 902 Automatic Analyzer (Roche Diagnostics, Indianapolis, IN). Serum lipoprotein cholesterol levels were analyzed by a combination of heparin-calcium precipitation and agar-agarose gel electrophoresis procedures. Plasma glucose levels were measured as part of a multiple chemistry profile (SMA20; Laboratory Corporation of America, Burlington, NC). A commercial radioimmunoassay kit was used for measuring plasma immunoreactive insulin levels (Phadebas; Pharmacia Diagnostics, Piscataway, NJ). Consistent assay kits were used for childhood insulin measurements at both baseline and follow-up surveys.

Adult MetS was defined according to NCEP ATP-III guidelines^37^. These criteria included: (1) abdominal obesity (WC ≥ 102 cm for men and ≥88 cm for women); (2) hypertriglyceridemia (fasting triglycerides ≥150 mg/dL, or taking cholesterol lowering medication); (3) low HDL-C (<40 mg/dL for men and <50 mg/dL for women, or taking cholesterol lowering medication); (4) high blood pressure (SBP/DBP ≥ 130/85 mmHg or taking antihypertensive medication); (5) hyperglycemia (fasting glucose ≥100 mg/dL or taking diabetic medication). Based on American Diabetes Association criteria, T2DM was defined as having fasting plasma glucose ≥126 mg/dL or glucose ≥126 mg/dL before taking diabetic medication, and impaired fasting glucose (IFG) was defined as 100~125 mg/dL of fasting plasma glucose.

### Statistical Methods

Analyses of covariance were performed using generalized linear models to test differences in study variables between race and gender groups. The study design of longitudinal changes of BMI and insulin measured at two time points in childhood is typically a cross-lagged panel design. The cross-lagged panel analysis is a form of path analysis that simultaneously examines reciprocal, longitudinal relationships among a set of inter-correlated variables^19^,^20^. A simplified, conceptual version of the model is presented in Fig. 1. The path with β_1_ describes the effect of baseline insulin on subsequent BMI, and the path with β_2_ describes the effect of baseline BMI on subsequent insulin. Prior to cross-lagged path analysis, the baseline and follow-up values of BMI and insulin were adjusted for age and gender by regression residual analyses and then standardized with Z-transformation (mean = 0, SD = 1) by race groups. The four variables in childhood generated six pair-wise observed correlations, and five correlations in Fig. 1 were used to estimate β_1_ and β_2_ because the connecting paths through the follow-up BMI-insulin correlation are not illegal according to the path analysis rules. Pearson correlation coefficients of the Z-transformed quantitative variables of BMI and insulin at baseline and follow-up were calculated, with adjustment for follow-up years. The cross-lagged path coefficients (β_1_ and β_2_) in the path diagram in Fig. 1 were estimated simultaneously based on the correlation matrix using the maximum likelihood method by the program LISREL 8.52. The validity of model fitting was indicated by RMR and CFI^38^,^39^. The difference between Pearson correlation coefficients and between β_1_ and β_2_ derived from the standardized variables (Z-scores) was tested using Fisher’s Z-test as described in our previous studies^18^,^40^. Although the significance of individual β_1_ or β_2_ suggests the directionality, a significant difference between β_1_ and β_2_ provides stronger evidence for a temporal relationship between BMI and insulin. Furthermore, cross-lagged analyses of childhood BMI and insulin were conducted by adult MetS vs non-MetS groups and adult hyperglycemic vs normoglycemic groups, with adjustment for age, race, gender, and follow-up years.

Once the temporal relationship between childhood BMI and insulin had been established, causal mediation models were constructed to examine whether the association of childhood BMI with adult MetS and T2DM was mediated by childhood insulin. Childhood baseline BMI was predictor variable (X); childhood follow-up insulin was mediator (M); MetS or T2DM were outcome variables (Y). In general, there are four steps for mediation analyses: (1) showing that the predictor variable determines the outcome (Model Y = β_Tot_ X) (β_Tot_ = total effect); (2) showing that the predictor variable affects the mediator (Model M = β_1_ X) (β_1_ = indirect effect 1); (3) showing that the mediator determines the outcome controlling for the predictor (Model Y = β_2_ M + β_Dir_ X) (β_2_ = indirect effect 2, β_Dir_ = direct effect); (4) calculating the proportion of mediation: mediation effect (%) = (β_1_ × β_2_/β_Tot_) × 100%. Mediation analysis was performed using R package *mediation*^41^, adjusted for age, race, gender, and follow-up years.

## Additional Information

**How to cite this article**: Zhang, T. *et al*. Long-term Impact of Temporal Sequence from Childhood Obesity to Hyperinsulinemia on Adult Metabolic Syndrome and Diabetes: The Bogalusa Heart Study. *Sci. Rep.* **7**, 43422; doi: 10.1038/srep43422 (2017).

**Publisher's note:** Springer Nature remains neutral with regard to jurisdictional claims in published maps and institutional affiliations.

## Supplementary Material

Supplementary Information

## Figures and Tables

**Table 1:** Descriptive data of study variables in childhood and adulthood by race and gender.

	Whites (N = 609)		Blacks (N = 339)		P-values^a^	
	Males	Females	Males	Females	Males	Females
N	246	363	125	214		
Baseline in Childhood						
Age (year)	10·6 (3·1)	10·5 (3·1)	10·3 (3·4)	10·3 (3·1)	0·465	0·525
BMI (kg/m^2^)	18·5 (3·9)	18·5 (4·1)	18·2 (3·9)	18·7 (4·3)	0·383	0·524
Insulin (μU/mL)	10·1 (10·0)	11·1 (8·6)	10·1 (8·7)*	12·5 (7·9)	0·988	0·064
Follow-up in Childhood						
Age (year)	15·8 (2·3)	15·8 (2·4)	16·1 (2·3)	15·9 (2·5)	0·282	0·560
BMI (kg/m^2^)	22·4 (4·7)	22·1 (4·9)	22·9 (5·3)	23·8 (6·0)	0·341	<0·001
Insulin (μU/mL)	11·0 (6·2)	11·6 (7·4)	11·4 (7·5)**	14·5 (11·3)	0·556	<0·001
Adulthood						
Age (year)	31·7 (7·2)*	30·4 (7·6)	29·3 (7·8)	29·5 (7·3)	0·002	0·157
Follow-up years^b^	15·9 (6·5)*	14·6 (6·9)	13·2 (7·2)	13·6 (7·0)	<0·001	0·084
BMI (kg/m^2^)	29·1 (6·4)*	27·6 (7·8)	28·6 (7·0)**	31·2 (8·8)	0·457	<0·001
Insulin (μU/mL)	12·5 (9·8)	11·4 (7·4)	12·3 (9·5)*	15·0 (10·0)	0·851	<0·001
WC (cm)	98·0 (17·3)**	85·5 (18·2)	91·8 (18·0)	92·6 (19·4)	0·001	<0·001
Systolic BP (mmHg)^c^	115·9 (10·5)**	107·4 (9·3)	121·2 (16·1)**	111·4 (12·0)	<0·001	<0·001
Diastolic BP (mmHg)^c^	78·2 (8·4)**	72·9 (7·9)	78·7 (13·1)**	73·8 (9·9)	0·693	0·213
HDL-C (mg/dL)^c^	41·5 (10·2)**	48·6 (12·5)	51·5 (16·2)	51·5 (13·1)	<0·001	0·011
Triglycerides (mg/dL)^c^	145·7 (110·4)**	110·8 (63·4)	107·0 (79·1)**	79·2 (36·2)	0·001	<0·001
Glucose (mg/dL)^c^	85·3 (10·6)**	81·5 (9·5)	89·0 (27·5)*	83·8 (17·9)	0·063	0·055
MetS, n (%)	61 (24·8)	69 (19·0)	25 (20·0)	33 (15·4)	0·366	0·328
IFG, n (%)	17 (7·1)	13 (3·7)	8 (6·9)	9 (4·4)	1·000	0·833
T2DM, n (%)	6 (2·6)	10 (2·9)	9 (7·7)	11 (5·4)	0·056	0·206

Continuous variables are presents as means (SD).

BMI = body mass index; WC = Waist circumference; BP = blood pressure; HDL-C = high-density lipoprotein cholesterol; MetS = metabolic syndrome; IFG = impaired fasting glucose; T2DM = type 2 diabetes.

^a^P-values for race difference in continuous metabolic variables adjusted for age.

^b^Follow-up period from the time point of the last childhood survey.

^c^Individuals who took medications were excluded.

Gender difference within race: *p < 0·05; **p < 0·01.

**Table 2:** Cross-lagged path coefficients of BMI and insulin in total, whites/blacks, MetS/non-MetS, and hyperglycemia/normoglycemia groups.

	Path Coefficients					Goodness-of-fit	
	Insulin → BMI		BMI → Insulin		p-value^a^		
	β_1_	p	β_2_	p		RMR	CFI
Total (n = 948)	−0·023	0·207	0·326	<0·001	<0·001	0·062	0·904
Whites (n = 609)	−0·033	0·136	0·298	<0·001	<0·001	0·064	0·895
Blacks (n = 339)	−0·010	0·754	0·374	<0·001	<0·001	0·056	0·921
p-value^b^	0·735		0·207				
Non-MetS (n = 760)	−0·007	0·750	0·190	<0·001	<0·001	0·061	0·908
MetS (n = 188)	−0·109	0·004	0·510	<0·001	<0·001	0·060	0·903
p-value^c^	0·212		<0·001				
Normoglycemia (n = 865)	−0·027	0·167	0·285	<0·001	<0·001	0·065	0·896
IFG (n = 47)	−0·034	0·566	0·526	<0·001	0·004	0·018	0·998
T2DM (n = 36)	−0·019	0·847	0·567	<0·001	0·007	0·062	0·889
Hyperglycemia (n = 83)	−0·032	0·565	0·503	<0·001	<0·001	0·047	0·943
p-value^d^	0·964		0·059				
p-value^e^	0·964		0·048				
p-value^f^	0·966		0·026				

BMI = body mass index; MetS = metabolic syndrome; IFG = impaired fasting glucose; T2DM = type 2 diabetes; RMR = Root mean square residual; CFI = Comparative fit index.

All β_1_ and β_2_ values were estimated by adjustment for age, gender, race and follow-up years.

^a^P-value for difference between β_1_ and β_2._

^b^P-value for difference in β_1_ and β_2_ between whites and blacks.

^c^P-value for difference in β_1_ and β_2_ between MetS and non-MetS groups.

^d^P-value for difference in β_1_ and β_2_ between IFG and normoglycemia groups.

^e^P-value for difference in β_1_ and β_2_ between T2DM and normoglycemia groups.

^f^P-value for difference in β_1_ and β_2_ between hyperglycemia and normoglycemia groups.
