# Supplementary material for: Long-term Impact of Temporal Sequence from Childhood Obesity to Hyperinsulinemia on Adult Metabolic Syndrome and Diabetes: The Bogalusa Heart Study
Source: Sci Rep. 2017 Feb 23;7:43422. doi: 10.1038/srep43422 (PMC5322533; doi:10.1038/srep43422)

Supplementary Information

Long-term Impact of Temporal Sequence from Childhood Obesity to Hyperinsulinemia on Adult Metabolic Syndrome and Diabetes: The Bogalusa Heart Study

Tao Zhang[1,2], Huijie Zhang[2,3], Ying Li[2,4], Shengxu Li[2], Camilo Fernandez[2], Lydia Bazzano[2], Jiang He[2], Fuzhong Xue[1,*], Wei Chen[2,*]

[1] Department of Biostatistics, School of Public Health, Shandong University, Jinan, China

[2] Department of Epidemiology, School of Public Health and Tropical Medicine, Tulane University, New Orleans, LA, USA

[3] Department of Endocrinology and Metabolism, the First Hospital of Xiamen, Xiamen University, Xiamen, China

[4] Department of Nutrition and Food Hygiene, School of Public Health, Harbin Medical University, Harbin, China

Short Title: Childhood BMI/Insulin and Adult Hyperglycemia

***Correspondence & Reprints:**

Wei Chen, MD, PhD

1440 Canal Street, Room 1504G, New Orleans, LA 70112

Tel: (504) 988-7611; Fax: (504) 988-7194

Email: wchen1@tulane.edu

Fuzhong Xue, MD, PhD

PO Box 100, 44 Wenhua Xi Road, Jinan 250012, China.

Tel: +86-531-88380280, Fax: +86-531-88380280

Email: xuefzh@sdu.edu.cn

Authors have no conflict of interest.

SupplementTable S1. Pearson correlation coefficients* between childhood BMI and insulin

		Baseline BMI	Baseline insulin	Follow-up BMI
Total (n=948)	Baseline insulin	0.335		
	Follow-up BMI	0.828	0.257	
	Follow-up insulin	0.346	0.169	0.481
Whites (n=609)	Baseline insulin	0.316		
	Follow-up BMI	0.835	0.234	
	Follow-up insulin	0.301	0.105	0.455
Blacks (n=339)	Baseline insulin	0.369		
	Follow-up BMI	0.817	0.293	
	Follow-up insulin	0.429	0.287	0.528
Non-MetS (n=760)	Baseline insulin	0.236		
	Follow-up BMI	0.803	0.183	
	Follow-up insulin	0.204	0.103	0.356
MetS (n=188)	Baseline insulin	0.452		
	Follow-up BMI	0.851	0.298	
	Follow-up insulin	0.500	0.208	0.618
Normoglycemia (n=865)	Baseline insulin	0.299		
	Follow-up BMI	0.818	0.220	
	Follow-up insulin	0.299	0.133	0.449
IFG (n=47)	Baseline insulin	0.633		
	Follow-up BMI	0.915	0.559	
	Follow-up insulin	0.396	0.128#	0.424
T2DM (n=36)	Baseline insulin	0.362		
	Follow-up BMI	0.818	0.280#	
	Follow-up insulin	0.607	0.316#	0.698
Hyperglycemia (IFG and T2DM, n=83)	Baseline insulin	0.527		
	Follow-up BMI	0.865	0.433	
	Follow-up insulin	0.527	0.310	0.604

BMI=body mass index; MetS=metabolic syndrome; IFG=impaired fasting glucose; T2DM=type 2 diabetes
* Partial Pearson correlations were calculated by adjusting for age, gender, race and follow-up years (without race in race groups).

All correlation coefficients are significantly different from zero ($p<0.05$) except those marked by #.

Supplement Table S2. Cross-lagged path coefficients (95% confidence intervals)of childhood BMI and insulin by tertile groups of follow-up years in childhood, with adjustment for covariates*

Follow-up years during childhood	Insulin→BMI			BMI→Insulin		p-value†
	β_1	95%CI		β_2	95%CI	
2.0~3.5 years (n=316)	-0.037	-0.084~0.009		0.362	0.251~0.473	<0.001
3.6~5.9 years (n=316)	0.013	-0.046~0.071		0.278	0.168~0.389	<0.001
6.0~14.7 years (n=316)	-0.056	-0.136~0.024		0.319	0.211~0.428	<0.001

* Covariates included age, gender, race and follow-up years.

†p-values for difference between $\beta_1$andβ_2.

Supplement Table S3. Cross-lagged path coefficients (95% confidence intervals, CI) of childhood BMI and insulin in MetS/non-MetS, and hyperglycemia/normoglycemiagroups, with adjustment for covariates*, by tertile groups of number offollow-up years from the second measurement in childhood to adulthood

Follow-up years	Insulin→BMI			BMI→Insulin		p-value†
	β_1	95% CI		β_2	95% CI	
**Non-MetS(n=760)**						
3.0~9.1 years (n=254)	0.041	-0.042~0.123		0.308	0.187~0.429	<0.001
9.2~16.0years (n=253)	-0.053	-0.130~0.023		0.121	-0.005~0.248	0.051
16.1~29.8 years(n=253)	-0.036	-0.099~0.027		0.204	0.082~0.327	<0.001
**MetS (n=188)**						
3.1~16.0 years (n=63)	-0.125	-0.31~0.060		0.526	0.253~0.798	<0.001
16.1~23.4years (n=63)	-0.032	-0.161~0.098		0.575	0.319~0.831	<0.001
23.5~30.2 years(n=62)	-0.203	-0.330~-0.076		0.526	0.308~0.744	<0.001
**Normoglycemia (n=865)**						
3.0~10.1 years (n=289)	0.019	-0.062~0.100		0.307	0.192~0.422	<0.001
10.2~16.8 years (n=288)	-0.078	-0.144~-0.011		0.236	0.115~0.356	<0.001
16.9~30.2 years(n=288)	-0.042	-0.098~0.015		0.342	0.230~0.453	<0.001
**Hyperglycemia (n=83)**						
4.1~17.0 years (n=28)	0.126	-0.094~0.346		0.341	-0.079~0.761	0.419
17.1~23.9years (n=28)	-0.070	-0.264~0.125		0.474	0.063~0.884	0.039
24.0~28.7 years(n=27)	-0.074	-0.296~0.149		0.749	0.512~0.986	<0.001

* Covariates included age, gender, race and follow-up years.

†p-values for difference between β_1 and β_2

Supplement Figure S1. Cross-lagged analysis models of childhood BMI and insulin in MetS and non-MetS groups, adjusted for race, age, gender, and follow-up years

β_1, β_2 = cross-lagged path coefficients; r_1 = synchronous correlations; r_2, r_3 = tracking correlations; R^2 = variance explained;

Goodness-of-fit: RMR=0.060 and CFI=0.903 for MetS; RMR=0.061 and CFI=0.908 for non-MetS

Coefficients different from 0: * p<0.01

†, p<0.001 for difference in β_1s between MetS and non-MetS groups

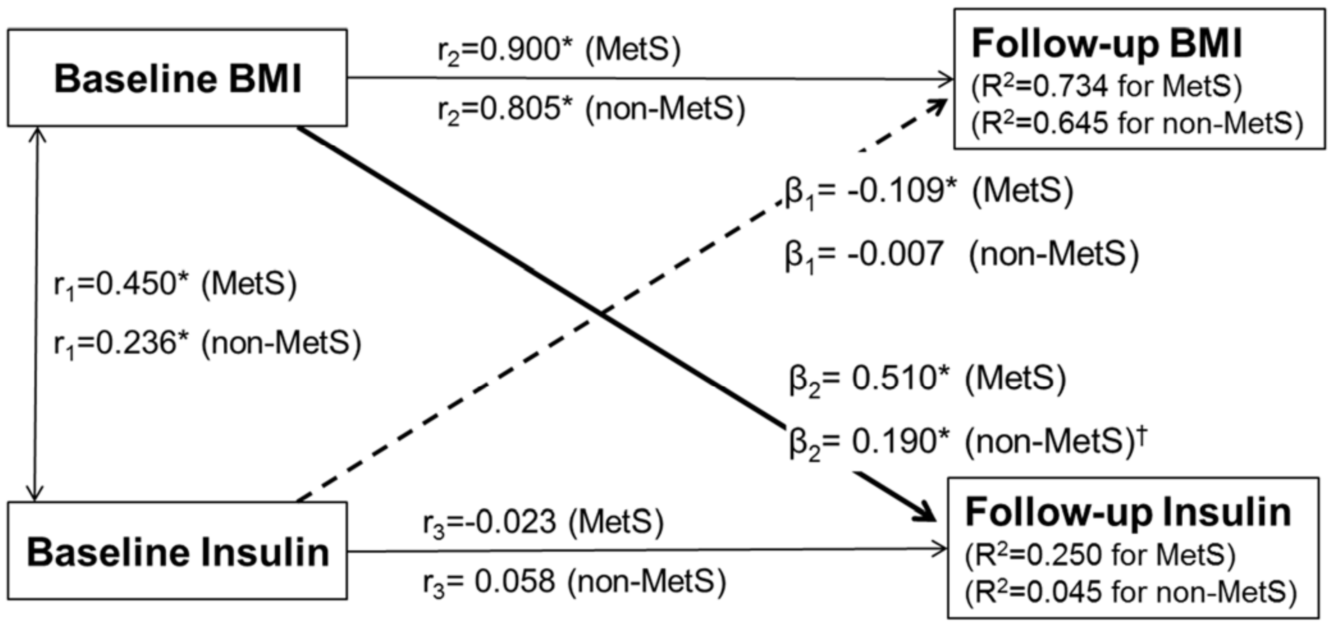

Supplement Figure S2. Cross-lagged analysis models of childhood BMI and insulin in normoglycemic and hyperglycemic groups, adjusted for race, age, gender, and follow-up years

β_1, β_2 = cross-lagged path coefficients; r_1 = synchronous correlations; r_2, r_3 = tracking correlations; R^2 = variance explained

Goodness-of-fit: RMR=0.047 and CFI=0.943 for hyperglycemia; RMR=0.065 and CFI=0.896 for normoglycemia

Coefficients different from 0: * p<0.01

†, p<0.001 for difference in β_1s between normoglycemic and hyperglycemic groups

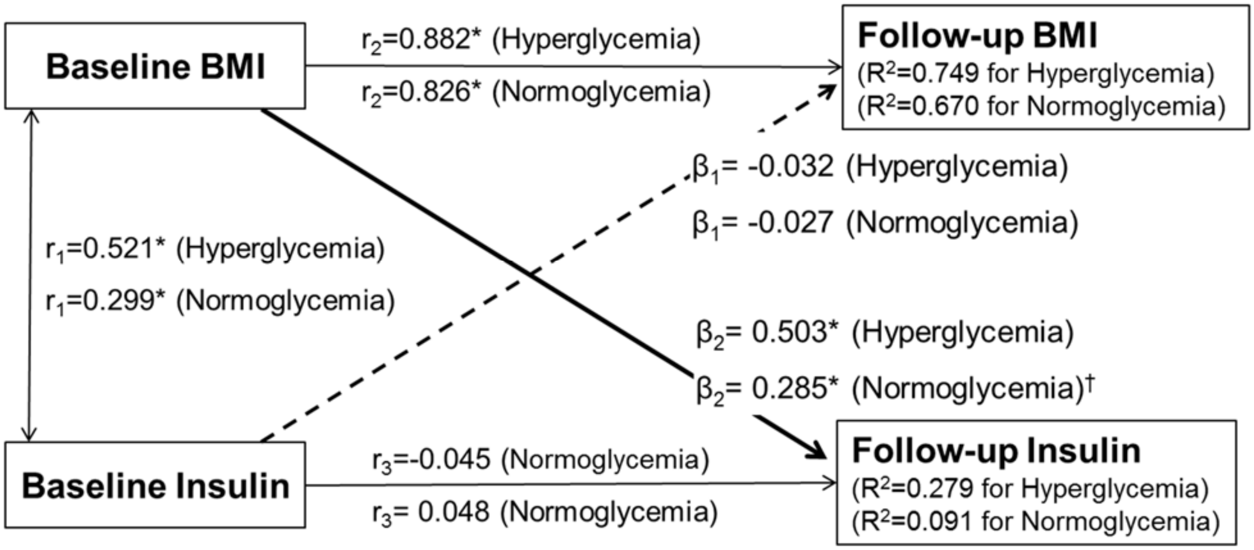

Supplement: Supplementary Information [file srep43422-s1.pdf]
